# The promotion of oral health within the Healthy School context in England: a qualitative research study

**DOI:** 10.1186/1472-6831-9-3

**Published:** 2009-01-15

**Authors:** Emma Stokes, Cynthia M Pine, Rebecca V Harris

**Affiliations:** 1 Department of Dental Public Health, Wirral Primary Care Trust Dental Services, Bromborough, CH62 4NH, UK; 2 Faculty of Health and Social Care, Allerton Building, University of Salford, Salford, M6 6EU, UK; 3 School of Dental Sciences, Liverpool University Dental Hospital and School of Dentistry, Liverpool, L3 5PS, UK

## Abstract

**Background:**

Healthy Schools programmes may assist schools in improving the oral health of children through advocating a common risk factor approach to health promotion and by more explicit consideration of oral health. The objectives of this study were to gain a broad contextual understanding of issues around the delivery of oral health promotion as part of Healthy Schools programmes and to investigate the barriers and drivers to the incorporation of oral health promoting activities in schools taking this holistic approach to health promotion.

**Methods:**

Semi-structured telephone interviews were carried out with coordinators of Healthy Schools programmes in the Northwest of England. Interview transcripts were coded using a framework derived from themes in the interview schedule.

**Results:**

All 22 Healthy Schools coordinators participated and all reported some engagement of their Healthy Schools scheme with oral health promotion. The degree of this engagement depended on factors such as historical patterns of working, partnerships, resources and priorities. Primary schools were reported to have engaged more fully with both Healthy Schools programmes and aspects of oral health promotion than secondary schools. Participants identified healthy eating interventions as the most appropriate means to promote oral health in schools. Partners with expertise in oral health were key in supporting Healthy Schools programmes to promote oral health.

**Conclusion:**

Healthy Schools programmes are supporting the promotion of oral health although the extent to which this is happening is variable. Structures should be put in place to ensure that the engagement of Healthy Schools with oral health is fully supported.

## Background

The World Health Organisation (WHO) advocates using Health Promoting Schools/Healthy Schools (terms used interchangeably in this paper) to promote general and oral health [1,2]. Healthy Schools are established worldwide as mechanisms for improving the health of school communities by supporting the health education curriculum through the school ethos and environment [3,4].

WHO guidance on Health Promoting Schools has been translated into policy in many countries. In England, the National Healthy Schools Programme (NHSP) provides strategic leadership to local Healthy School programmes (LHSPs) by endorsing a whole-school approach focused on common risk factors (CRFs); risk factors such as diet, tobacco and exercise, which are shared by the major non-communicable diseases [5]. Current thinking supports the promotion of oral health through this approach since some of the main aetiological factors of oral diseases such as dental caries (high frequency and amount of sugar intake) are well-recognised as also being implicated in other conditions such as diabetes [6]. Risk factors for oral cancer are common to other types of cancer, and accident prevention programmes can prevent trauma to teeth and oral tissues.

The NHSP requires schools to achieve 'Healthy School' status by addressing the CRFs through focusing on four strands [7]:

• ***Personal, Social & Health Education ***(including drugs, tobacco, alcohol and sex and relationships education)

• ***Healthy Eating***

• ***Physical Activity***

• ***Emotional Health & Wellbeing***

Oral health is not discussed within the NHSP, although the English oral health strategy suggests that oral health should be promoted using the CRF approach within settings such as Healthy Schools [8]. LHSPs may not identify oral health as an important issue. Whether oral health is being promoted by the English NHSP has not been studied, although a small number of studies have been carried out in other national contexts.

A recent study in Scotland reported an association between Healthy School status and twice-daily toothbrushing for some children attending Healthy Schools in deprived areas [9]. The authors analysed data from the Health Behaviour in School-Aged Children Questionnaire using multilevel logistic regression. Schools were classified into two categories: having/working towards a Health Promoting School award or not having/not working towards a Health Promoting School award. No attempt was made to determine which schools-based activities may have influenced this difference in behaviour.

A study in Brazil has also reported oral health benefits for children attending schools engaging with the Health Promoting Schools philosophy [10]. Schools were categorised as being either supportive or non-supportive in terms of their engagement. Engagement was measured according to schools' implementation of health promoting strategies. Children in supportive schools had better and more homogenous health outcomes in terms of caries levels and dental trauma. Since none of the schools in the study had a formal dental health education programme, the differences observed could be attributed to the effect of a CRF approach in promoting oral health.

Whilst these studies point towards Healthy Schools having a positive impact on oral health, nothing is known about *how *the Healthy Schools programme translates into interventions influencing oral health. Therefore the research questions being addressed were:

What are the areas of the Healthy Schools programme which might impact on oral health? To what extent are these areas pursued within Healthy Schools programmes in the North-West of England? What are the barriers and drivers to the incorporation of oral health promoting activities within Healthy Schools programmes?

## Methods

Approval to carry out the study was obtained from the Central Office for Research Ethics Committees (COREC) (06/MRE/63), the Department of Health and employers of participants.

### Study design

The English NHSP is organised into 9 regions. The North-West region incorporates 2 large conurbations and some rural areas. Parts of the North-West region are among the most deprived areas in England, others are among the most affluent . Each region of the NHSP consists of several LHSPs that operate as partnerships between local education and health agencies. Coordinators of the 22 LHSPs in the North-West of England (Local Healthy School Coordinators) were identified as key informants for this study. These individuals have responsibility for managing LHSPs and as such, were considered to have the potential to provide both strategic and practical insights for the current study.

A qualitative rather than quantitative study design was selected because of the lack of previously reported data in this field. Semi-structured interview rather than focus group methodology was selected because of the difficulty in arranging for the participants to gather in one place. Interviews can also assist respondents in speaking freely [11]. The interviews were semi-structured to allow flexible data collection so that emergent issues could be explored alongside discussion of the key themes [12].

### Development of the interview schedule

Oral health promoting activities suitable for incorporation into the NHSP were identified from a literature search, followed by consultation with a range of senior personnel working in the field of dental public health by means of a questionnaire. A panel with expertise in the field of oral health promotion (OHP) reviewed the activities identified and thus finalised the interview schedule (Table 1).

**Table 1:** Interview schedule for telephone interviews with local Healthy Schools programme coordinators

**Preamble**
Introduction of interviewer
Confirmation of interviewees name
Confirmation that interviewee is happy for interview to be taped
Explanation of research
Confirmation of interview confidentiality and anonymity in reporting the data
Confirmation of the interview length

**Questions**

I'd like to start by asking you your feelings about oral health being promoted as part of an intervention like Healthy Schools?
*Prompts:*
*What do you see as the relationship between oral health and general health?*
*Who do you think should have responsibility for promoting oral health in schools?*

Are there any oral health promoting activities that you think could be incorporated within a Healthy Schools programme?
*Prompts:*
*What are the differences between what would be appropriate or possible in a primary or a secondary school?*
*Are you aware whether oral health is covered in the curriculum?*
*Are you aware whether staff receive training to teach oral health?*

Do you know whether oral disease is a particular problem in the local area covered by your Healthy Schools scheme?
*Prompt:*
*What is the extent of this problem?*

Are there any members of your team who include oral health within their remit?
*Prompts:*
*Who are they and what is their role?*
*Have you or they any particular experience in any aspects of oral health promotion?*

Does your local area have its own Healthy Schools manual? If so, is oral health explicitly or implicitly discussed within it?
*Prompts:*
*Where and how is it discussed?*
*Are you aware of any local directives that support the promotion of oral health in Healthy Schools?*
*Are you aware of any national directives that support the promotion of oral health in Healthy Schools?*

Do you have any local partners that have a role in supporting oral health promotion within schools?
*Prompts:*
*What is the current activity of these partners?*
*Is this activity carried out in all schools or selected schools? If selected schools, how are these selected?*
*Are parents or the wider community involved in any school oral health initiatives?*
*Are you aware of any oral health promotion programmes for school staff?*

Are you aware of any schools-based oral health promotion that takes place outside the Healthy Schools umbrella in your area?
*Prompt:*
Perhaps carried out by the Community Dental Service in schools that are not involved with the Healthy Schools scheme?

Does consideration of the safety of the physical environment of the school environment form part of the local Healthy Schools programme?
*Prompt:*
*Would schools have a policy that covers the safety of the school environment?*

Is the local Healthy Schools scheme promoting the use of cycle helmets and mouthguards for sports?
*Prompt:*
*Would schools have a trauma and emergency care policy?*

Are you aware of any schools that have an oral health policy or an oral health education policy?
*Prompt:*
*Or would these be covered by more general policies?*

In your opinion, what influences how and whether local Healthy Schools schemes engage with oral health promotion?

I now want to ask you some more about the activities that are perhaps more integral to the national Healthy Schools scheme and that could promote oral health.

Are schools involved with the local Healthy Schools programme expected to have a healthy eating policy?
*Prompts*
*Are they expected to follow this through by having healthy foods and drinks available?*
*What about sugar-free foods and drinks?*
*And drinking water?*
*Would they be expected to try and control outside vendors?*
*Do schools or the local area have a SNAG (School Nutrition Action Group)*

What sort of involvement with anti-smoking initiatives are Healthy Schools expected to have?
*Prompts:*
*What about school smoking and tobacco policy?*
*Or school antismoking programmes?*

How about involvement with alcohol programmes?
*Prompts:*
*Would schools have an alcohol policy?*
*And alcohol programmes?*

To what extent is student participation a part of the local Healthy Schools scheme?

Could I ask finally about the numbers of schools in your area involved with the Healthy Schools programme?
*Prompts:*
*What percentage of these has achieved Healthy School status?*
*What percentage of these are primary schools?*
*What percentage of these are secondary schools?*

**Concluding comments**

Would it be useful for you to have a copy of the report and recommendations from this study?
Thank you for your time. If I find that I need further clarification on any of the issues that we have talked about today, would it be OK to contact you again?

The schedule contained questions around three themes:

*A. The concept of promoting oral health as part of Healthy Schools*

*B. The delivery of OHP in Healthy Schools*

*C. Drivers for oral health to be promoted as part of Healthy Schools*

### Procedure

Coordinators of the 22 LHSPs in the Northwest of England were mailed to invite them to participate and to give them information about the study. Participants who did not reply were contacted by telephone and/or email to establish whether they were willing to take part. All 22 LHSP coordinators consented to participate in the study. Participants were assured that their responses would be anonomised. Interviews were audio-taped and transcribed.

### Data analysis

A coding framework based on the themes in the interview schedule was designed. Transcripts were examined manually to identify codes using thematic content analysis and a system of constant comparison [13,14]. This involved passing through the data several times, making comparisons and connections until no further codes were identified and the data was considered to be saturated. Ten codes were identified and categorised within the three main themes used in the interview schedule (Table 2). Transcripts were coded with the final set of codes using NVIVO software (QSR International). Coding was carried out by the interviewer (ES) and was verified by a second rater. Differences in opinion were resolved by consensus discussion.

**Table 2:** Coding framework used for the analysis of the interview transcripts

**Theme A: The concept of promoting oral health as part of Healthy Schools**
**Codes:**
i. The appropriateness of promoting oral health as part of Healthy Schools
ii. The relationship between oral and general health
iii. Responsibility for oral health promotion in Healthy Schools
**Theme B: The delivery of oral health promotion in Healthy Schools**
**Codes:**
iv. Main areas of NHSP
v. The whole school approach
vi. Organisation
vii. Challenges

**Theme C: Drivers for oral health to be promoted in Healthy Schools**
**Codes:**
viii. Levels of oral disease in the local area
ix. Local and national directives
x. Historical ways of working

### Study quality, reproducibility, reliability, validity and generalisability

This study referred to qualitative research review guidelines to ensure its quality [15]. Systematic processes and full methodological accounts were used to enhance reproducibility [16]. Reliability was ensured, and the potential impact of the researcher also being the interviewer minimised, through investigator triangulation in the coding, with the second coder not being otherwise engaged with the study [17]. Disagreements were settled through consensus discussion. It was difficult to check the validity of the study using data triangulation because of lack of previously collected data in the field [16,17]. Findings from this study will be tested for validity in future planned research in this field. A degree of validation was provided through the inclusion of 'negative' cases [16]. The high response rate assisted generalisability [18].

## Results

### Theme A: The concept of promoting oral health as part of Healthy Schools

#### The appropriateness of promoting oral health as part of Healthy Schools (i)

All the respondents felt that it was appropriate to promote oral health within Healthy Schools. All respondents placed OHP within the Healthy Eating strand of the NHSP. Six respondents also recognised the appropriateness of locating OHP within other strands of the programme:

(numbers in brackets after each quote refer to the participant)

*'it goes hand in hand with the Healthy Eating standard' *(9)

*'It's also an important part of the emotional health and ... the sex and relationship education' *(22)

#### The relationship between oral and general health (ii)

All respondents felt that oral and general health were linked. Three participants conceptualised oral health as an indicator of general health:

*'the health of the gums etc is an indicator in terms of heart disease' *(11)

Other respondents felt that there was no distinction between oral and general health for the purposes of promoting oral health within Healthy Schools. There was also recognition that oral health had historically been separated from general health, but that it should now be included as part of general health, and the Healthy School approach was therefore a positive thing in this context:

*'I think the key thing is ... the linkage. Oral health has sat on its own for so long' *(9)

#### Responsibility for OHP in Healthy Schools (iii)

Eight participants gave schools or school nurses the responsibility for OHP, others felt that the responsibility lay solely with specialist OHP teams or jointly between Healthy Schools teams, schools and OHP teams:

*'it's part of what schools teach young people' *(10)

*'It's got to be a partnership thing' *(12)

Many respondents acknowledged the necessity for the involvement of personnel with specialist oral health expertise.

### Theme B: The delivery of OHP in Healthy Schools

#### Main areas of NHSP (iv)

This code categorised oral health promoting activities within the main strands of the NHSP.

##### Healthy eating

All participants reported that schools addressing healthy eating as part of their Healthy Schools work were expected to have a healthy eating policy and to be acting on that policy. Irrespective of engagement with Healthy Schools, all schools were expected to conform to minimum nutritional standards [19]. There was variability in how LHSPs were requiring schools to achieve the healthy eating standard; for example, only some participants had made the guidance explicitly tooth-friendly.

##### Personal social and health education (PSHE)

Formal oral health education and education about alcohol and tobacco fall within the PSHE strand (alcohol and tobacco are risk factors for oral cancer). Although participants did not tend to make unprompted links between tobacco and alcohol and oral health, once prompted they were aware of the relevance of targeting these areas to promoting oral health. Tobacco interventions were being directed through national legislation e.g. The Smoke-free (Premises and Enforcement) Regulations (2006), as well as through Healthy Schools. Healthy Schools programmes required schools to have policies covering tobacco, alcohol and drugs. School interventions and education around these topics were more variable with some participants reporting smoking cessation workers visiting schools. In other areas schools-based smoking cessation was viewed as being inappropriate because it was felt that smoking cessation should be instigated by an individual's desire to quit.

##### Emotional health and wellbeing

Nine respondents recognised the relevance of oral health to this Healthy Schools strand:

*'there's a very nice project ... about the effect of your teeth on your whole face, and your own personality ... that's about self-esteem as well' *(11)

##### Physical activity

Respondents were asked about two aspects of physical activity that could prevent oro-facial trauma: the use of cycle helmets and sports mouthguards. Cycle helmets were commonly being promoted either directly by the LHSP, or by their partners. Sports mouthguards were less commonly promoted and one respondent raised concerns raised about the appropriateness of promoting mouthguard use:

*'because of the chance of choking' *(16)

#### The whole school approach (v)

This code related OHP activities to the five core features of a whole school approach.

##### The taught curriculum

Participants were generally positive about oral health being taught within schools, with most expressing that it should be taught either within PSHE or science. Not all participants were aware of precisely what was taught in relation to oral health. Some respondents expressed concerns about timetabling constraints.

##### Physical school environment

The physical environment of the school has the potential to impact on oral health because of its links to health and safety, accident prevention and emotional health and wellbeing. The extent to which it was being considered differed between localities. Some areas considered the physical environment of their schools as an area for schools to address after they achieved 'Healthy School' status. Others conceptualised assessing the physical school environment either as a part of ensuring that schools were using the whole school approach or as linked to the emotional health and wellbeing strand of the NHSP.

##### Healthy policies

All the participants except one were unaware of schools in their area having specific oral health policies; it was generally felt to be more appropriate that references to oral health should be embedded within more general policies:

*'it's also explicit in in you know the whole school food policy which we've modelled for them ...erm which which sort goes through ... DMFT figures ... so its sets the context of ...why it is important about healthy eating' *(18)

##### Pupil participation

Pupil participation may impact on oral health through promoting psychological health and wellbeing. In one area pupil participation was being used to promote oral health specifically:

*'we ... use some of the older children to be peer educators ... we've trained the children ...and they then have gone to work with some of the nursery children' *(11)

##### Involvement of parents and staff

Involving parents and staff in Healthy School programmes is a vital part of the whole school approach. Parents were more commonly involved in OHP in the primary than the secondary sector:

*'we're ... trying to educate parents of primary school children ... we emphasize that a lot more ...than we do with secondary children' *(10)

#### Organisation (vi)

This code described the resources, personnel and partners involved in the delivery of OHP.

##### Personnel and partners

Personnel involved with OHP in Healthy Schools sometimes included individuals working as core members of local Healthy Schools teams. In the majority of areas personnel also included people working for partner agencies such as OHP teams, local sports partnership teams, school nurses and drug education teams. OHP teams are generally dental nurses, hygienists, and therapists trained in oral health education and employed by the local salaried dental service. Only one participant reported that the LHSP was not working in partnership with an OHP team. The role of local OHP teams varied considerably between areas:

*'the OH *(oral health) *team come in and run a programme ... supporting the curriculum ... they've done something in terms of leaflets on resetting teeth, if they've been knocked out they ... support the work right throughout all Key Stages' *(16)

*'they do like puppet plays as well ...around the theme of OH' *(4)

In one area, the issue of expertise was addressed differently; the Healthy School team had recognised that they had more expertise about delivering interventions in schools than the local OHP team. They worked with the OHP team to ensure that OHP messages were provided in the most appropriate ways:

*'oral health were working ... quite independently of us .... they were too focused on oral health ... there was actually a jarring happening so that when Health Promoting Schools were going in as well ... it was beginning to be seen as a bit of a nanny state .... so what we decided to do was because we had more expertise about delivering in schools ... is to make the health messages education friendly ... we started working more closely with oral health ... one of their workers actually joined our team ' *(22)

##### Targeting resources

OHP interventions were sometimes targeted to either the primary sector and/or to those schools with the worst dental health. One reason for targeting the primary sector related to lack of uptake by secondary schools although other areas targeted the primary sector because of the perceived importance of establishing healthy behaviours at a young age.

#### Challenges (vii)

This code described challenges identified in promoting oral health in Healthy Schools.

##### Freedom of choice

This problem emerged in relation to schools being able to choose the extent to which they engaged with their local Healthy Schools programme or with OHP interventions:

*'all our schools are signed up, it doesn't necessarily mean they're doing anything' *(2)

##### Lack of expertise

Seven respondents discussed that lack of expertise was a challenge in delivering OHP:

*'we don't go into the secondaries ... we haven't got enough medical knowledge' *(22)

##### Logistics

Many of the challenges in delivering OHP in Healthy Schools discussed by participants were logistical:

*'if you give them water in classes, they'll just mess about with it' *(10)

##### Mixed messages

Participants noted that there was evidence of mixed messages occurring in schools, for example with unhealthy food being served at some school events.

##### Outside vendors

The problem of outside vendors (ice cream vans at the school gates etc) was common to several localities. There was evidence of work going on to address the issue in six areas, by involving vendors themselves, trading standards and/or environmental health. Participants expressed a wish for guidance on this issue.

##### Profile of oral health

The low profile of oral health was discussed by twelve respondents as a potential barrier to promoting oral health in Healthy Schools. Interviewees cited the exclusion of oral health from National Healthy Schools guidance, although they did recognise that oral health may be promoted as part of the CRF approach, despite the lack of national guidance:

*'it's hard because there's no specific reference to it within Healthy Schools ... at the moment the focus is on Healthy Schools status' *(2)

*'it's almost implicit in the healthy eating work that you do. I don't think it's perhaps given as high a priority as it might do' *(18)

There were also comments made relating to the low profile of oral health on the local agenda:

*'oral health hasn't been targeted as a priority this time' *(2)

Respondents were also unsure if schools would make the link between Healthy Schools and oral health and recognised that this may depend on circumstances within individual schools.

##### The secondary sector

Problems identified specific to the secondary sector included accessing parents, engaging schools and the tight curriculum.

### Theme C: Drivers for oral health to be promoted as part of Healthy Schools

#### Levels of oral disease in the local area (viii)

Seventeen participants considered that oral disease in their area was a problem:

*'we've got the worst OH erm data in the country' *(8)

A few respondents did not think that oral disease was a local problem or recognised that although local levels of oral disease were high, the problem had not been prioritised:

*'as far as I'm aware it isn't' *(19) (area in the best quartile for oral health in the Northwest)

*'it hasn't been deemed to be *(a priority), .... *that's not saying that it isn't' *(2) (area in the second worst quartile for oral health in the North-West)

#### Local and national directives (ix)

In some areas oral health was a local priority:

*'we've specifically said good practice is linked to dental health' *(15)

Respondents were unaware of the existence of any national strategies or directives that would support the promotion of oral health as part of Healthy Schools:

*'we've not got any sort of directives, not sort of nationally' *(17)

#### Historical ways of working (x)

Historical ways of working emerged from the data as a way to explain how many local Healthy Schools programmes had come to be so involved with oral health:

*'we've always done it in this area' *(17)

## Discussion

Recent evidence of an association between periodontal disease and systemic diseases such as coronary heart disease and diabetes [20], has brought an increased focus on the importance of oral health in relation to general health. As noted by some of the participants in the study, oral health has often been considered as a separate entity from other health considerations. The Healthy Schools programme does not consider separate diseases as part of the overall strategy guiding the programme. The question then arises 'Are preventive programmes pertinent to preventing oral disease implemented as a result of the Healthy Schools programme, or do some gaps exist?' This study suggests that indeed there are some areas where oral health issues are fully addressed, but there are also other areas where oral health issues are only partly addressed; and full coverage of oral health within the Healthy Schools programme is often dependent on historical ways of working and input from specialised dental personnel working on health promotion and supporting the school activity.

When interpreting the data generated from evaluations of health promotion interventions it is difficult to disentangle the intervention being studied from other interventions [21]. In the case of Healthy Schools, this difficulty is amplified by the partnership approach embedded within the philosophy and by the possibility that schools who have not engaged with Healthy Schools programmes may be accessing other health promotion programmes and interventions. The results for example indicate that there are several other influences on policy and practice in schools such as legislation related to healthy eating and creating smoke-free environments. However, whilst being unable to conclude that oral health is or is not being promoted as a direct result of the Healthy Schools programmes, this study was able to demonstrate ways in which Healthy Schools programmes are promoting oral health.

Care is necessary in extrapolating the results of this study to wider national and international contexts. However, there is no evidence that the North-West region is different from other English Healthy School regions, in the way in which its Healthy Schools programmes engage with OHP. Further, because local Healthy Schools programmes work within the NHSP framework [7] and towards national targets, it would be reasonable to suggest that the results of research in one Healthy Schools region might be applicable to the national wider context. In terms of international applicability, this research provides data describing the practical aspects of using the CRF approach to promote oral health in school settings and describes issues related to the challenges of using this approach to promote oral health.

Results showed that some Healthy Schools programmes were relying largely on the promotion of oral health through the CRF approach, whilst others were more explicit in promoting oral health. Explicit OHP tended to be related to historical ways of working, and often involved input from local OHP teams using dental personnel. In some areas OHP teams were offering specific schools-based interventions. In other areas, OHP teams were more involved in providing advocacy and training, roles which have been recommended for oral health experts working within general health promotion frameworks [22-25]. The role of expert (dental) input is an important issue when considering promoting oral health using the CRF approach [22-25]. There was consensus among participants in this study that expert input was necessary. Expertise was valued in relation to raising the profile of oral health and providing input to schools, local programmes and strategic groups. Further research is needed to identify the most appropriate way to utilise oral health expertise within Healthy Schools programmes.

OHP was most often related to the healthy eating and PSHE strands of the NHSP. Whilst it is easy to envisage the positive impact of the promotion of healthy eating within the school on levels of dental caries, it is more difficult to see how Healthy Schools programmes might impact on levels of toothbrushing, as indeed they appear to [9]. It is well established that toothbrushing habits are related to general hygiene and cleanliness [26], and that that school satisfaction and self-esteem are related to more-than-once-a-day toothbrushing [27]. Perhaps emphasis within the Healthy Schools programme on building a positive environment for children has had some indirect benefits on their oral hygiene habits although less than half of the coordinators of the LHSPs in this study recognised the relevance of oral health to the emotional health and wellbeing strand of the NHSP.

## Conclusion

The integration of OHP within Healthy Schools programmes was occurring despite participants' lack of awareness of international and national guidance in relation to oral health [2,8]. Currently, although *local *Healthy School coordinators seem to have made linkages between Healthy Schools and oral health, Healthy Schools policy makers at a *national *level have excluded oral health from guidance. National guidance on OHP in Healthy Schools would legitimise the promotion of oral health in Healthy Schools and secure its future against the threats identified by participants in this study. The authors suggest a national document based on the WHO publication on OHP in Health Promoting Schools [2]. The authors also recommend further research to establish which methods are the most effective and appropriate ways of promoting oral health in Healthy Schools.

## Competing interests

The authors declare that they have no competing interests.

## Authors' contributions

ES designed the study, carried out the fieldwork and drafted the manuscript. CMP and RVH assisted in the design of the study and advised on the preparation of the manuscript. All the authors read and approved the final manuscript.

## Pre-publication history

The pre-publication history for this paper can be accessed here:


